# Temporal trends in emergency department volumes and crowding metrics in a western Canadian province: a population-based, administrative data study

**DOI:** 10.1186/s12913-020-05196-4

**Published:** 2020-04-26

**Authors:** Brian H. Rowe, Andrew McRae, Rhonda J. Rosychuk

**Affiliations:** 1 Department of Emergency Medicine, Faculty of Medicine & Dentistry, University of Alberta, Edmonton, Alberta T6G 2R7 Canada; 2 School of Public Health, University of Alberta, Edmonton, Alberta Canada; 3 Department of Medicine, University of Alberta, Edmonton, Alberta T6G 2R7 Canada; 4 Department of Emergency Medicine, University of Calgary, C231 Foothills Medical Centre, 1403 29 St NW, Calgary, Alberta T2N 2T9 Canada; 5 Department of Community Health Sciences, University of Calgary, Calgary, Alberta Canada; 6 Department of Pediatrics, Faculty of Medicine & Dentistry, University of Alberta, 3-524 Edmonton Clinic Health Academy, Edmonton, Alberta T6G 1C9 Canada; 7 Department of Mathematical and Statistical Sciences, University of Alberta, Edmonton, Alberta Canada; 8 Department of Statistics and Actuarial Science, Simon Fraser University, Burnaby, British Columbia Canada

**Keywords:** Emergency department, Crowding metrics, Admission, Patient flow

## Abstract

**Background:**

Emergency Department (ED) crowding is a pervasive problem, yet there have been few comparisons of the extent of, and contributors to, crowding among different types of EDs. The study quantifies and compares crowding metrics for 16 high volume regional, urban and academic EDs in one Canadian province.

**Methods:**

The National Ambulatory Care Reporting System (NACRS) provided ED presentations by adults to 16 high volume Alberta EDs during April 2010 to March 2015 for this retrospective cohort study. Time to physician initial assessment (PIA), length of stay (LOS) for discharges and admissions were grouped by start hour of presentation and facility. Multiple crowding metrics were created by taking the means, medians (PIA-M, LOS-M), and 90th percentiles of the hourly, ED-specific values. Similarly, proportion left against medical advice (LAMA) and proportion left without being seen (LWBS) were day and ED aggregated. Calculated based on the start of the presentation and the facility and for PIA and LOS. The mean, median, and 90th percentiles for the date and time ED-specific metrics for PIA and LOS were obtained. Summary statistics were used to describe crowding metrics.

**Results:**

There were 3,925,457 presentations by 1,420,679 adults. The number of presentations was similar for each sex and the mean age was 46 years. Generally, the three categories of EDs had similar characteristics; however, urban and academic/teaching EDs had more urgent triage scores and a higher percentage of admissions than regional EDs. The median of the PIA-M metric was 1 h23m across all EDs. For discharges, the median of the LOS-M metric was 3h21m whereas the median of the LOS-M metric for admissions was 10h08m. Generally, regional EDs had shorter times than urban and academic/teaching EDs. The median daily LWBS was 3.4% and the median daily LAMA was about 1%.

**Conclusions:**

Emergency presentations have increased over time, and crowding metrics vary considerably among EDs and over the time of day. Academic/teaching EDs generally have higher crowding metrics than other EDs and urgent action is required to mitigate the well-known consequences of ED crowding.

## Background

Emergency Department (ED) crowding occurs when “the demand for emergency services exceeds the ability of an emergency department to provide quality care within appropriate time frames” [1–3]. Many urban, high-volume, tertiary care EDs in North America continue to face a crowding crisis and the quality of care can suffer during any peak periods of demand. The consequences of ED crowding can be serious as patients may not receive time-sensitive interventions (e.g., reperfusion for myocardial infarction and stroke [4], analgesia for severe pain [5], treatments for severe infections [6], and advanced diagnostic imaging [7]), may leave without being seen by a physician (LWBS) or against medical advice (LAMA), and if critically ill, may have longer lengths of stay in hospital or greater hospital mortality [8].

ED crowding is believed to be influenced by multiple factors and in Canadian hospitals, the most important factor leading to ED crowding is the lack of in-patient beds for those presenting to the ED who need to be admitted (also called “access block”) [3]. The multiple influencing factors are related to aspects of patient flow and can be characterized by a conceptual model with input, throughput, and output [1]. Input, throughput, and output respectively refer to how patients arrive in the ED (e.g., ambulatory, via ambulance or referral), the processes of ED care, and their eventual disposition (e.g., LWBS/LAMA, discharge/admission, transfer, death). Multiple metrics have been defined to capture the input, throughput, and output aspects.

There is a paucity of literature on the distribution of ED crowding metrics in high volume EDs, including differences among rural, urban community and academic hospitals. In Canada, high volume EDs are known to experience the highest crowding pressure [9]. To address this knowledge gap, we used robust, population-based, administrative data to examine input, throughput and output metrics in 16 high volume EDs in Alberta, Canada. We compared metrics among ED type, and contrasted them against accepted national benchmarks for each metric [3]. Our primary objectives were to summarize the ED crowding metrics in the 16 high volume EDs overall and by ED category (regional, urban, academic/teaching). Our secondary objectives were to compare the proportions of ED presentations that met recommendations for input, throughput and output metrics, and to describe any temporal trends in the ED crowding metrics and achievement of recommended targets. We hypothesized that EDs were not regularly achieving nationally accepted wait time standards and that the sub-type EDs (regional, urban, and academic) would produce unique differences. We further anticipated observed differences among ED types and over time could lead to changes in resource allocation or operational policies to specifically target root causes of crowding that may differ between ED types.

## Methods

### Study design

This retrospective cohort study used a population-based health administrative database from the province of Alberta, Canada during April 1, 2010, to March 31, 2015. This study was approved by the University of Alberta Health Research Ethics Board. The funding organization (Alberta Health) had no input in the conduct and reporting of the study.

### Study setting and population

Alberta is a Canadian province with > 4 million residents and a uniform single-payer health system –the Alberta Health Care Insurance Plan (AHCIP) –that provides medically necessary health care. The Government of Alberta is the custodian of all administrative databases used in our study.

The study population consists of individuals aged ≥18 years who presented to the high volume EDs (> 50,000 presentations per year) during the study period. Visitors to the EDs who were not residents of Alberta at any time during the study period or who were not registered with the AHCIP were not included in the analyses; generally < 1% of all records would be from non-residents of Alberta [10]. The study focused on Alberta’s 16 highest volume EDs, classified into three categories: regional (*n* = 5), urban (*n* = 8), and academic/teaching (*n* = 3). The academic/teaching EDs see adult patients exclusively. Adult presentations were extracted from the National Ambulatory Care Reporting System (NACRS). Patients, hospital staff, and the public were not involved in or aware of this research.

### Study protocol

The NACRS database provides information on the dates and times related to an ED presentations, triage level, and disposition status. The date/time variables included the date/time of registration, date/time of triage, date/time of physician initial assessment, date/time of disposition decision, and date/time patient left the ED. The start of the ED presentation was set to be the minimum of the registration and triage dates and times. The start of the presentation was used to define fiscal year, month of year, weekday/weekend, and time of shift (daytime 08:01–16:00, evening 16:01–24:00, night 00:01–08:00). Triage level represents the urgency of ED care required by the individual and is based on the Canadian Emergency Department Triage and Acuity Scale (CTAS) [11, 12]. The triage codes are: resuscitation (1), emergency (2), urgent (3), semi-urgent (4), and non-urgent (5). Patients are given one of 10 disposition codes according to the way in which they are released from ED and we have grouped these as discharges, admissions, transfers, deaths, and left without completion of care (e.g., LAMA, LWBS).

The NACRS database also provides demographic and geographic data collected at the time of the ED presentation. Age is provided as age in years at date of ED presentation. Sex is either male, female, or other, since other gender details were not collected in the database. The health zone of residence (North, Edmonton, Central, Calgary, South) are reported according to where the person lived at the time of the ED presentation.

### Key outcome measures

We used recommended ED crowding metrics [3] based on all presentations made by patients to a facility for any condition: time to physician initial assessment (PIA), and length of stay (LOS) in the ED for discharges and admissions, percent LAMA, and percent LWBS. For all presentations that had dispositions other than left without completion of care, the time from ED presentation start (usually triage) to the first assessment by a physician was used to calculate PIA. Negative PIA were assigned a value of zero since in a critical situation it is acknowledged that physician assessment may occur before registration/triage [13]. The Canadian Association of Emergency Physicians (CAEP) standards were use throughout, and they recommend a median of 1 h and 90th percentile of 3 h as targets for PIA [3].

The ED LOS was calculated depending on the disposition: it was calculated as the time from the start of ED presentation until the time the disposition decision for discharged patients or time the patient departed the ED for an in-patient unit if patient was admitted [13]. For discharged patients, CAEP recommended median LOS of 4 h (90th percentile of 8 h) for CTAS 1/2/3 patients and 2 h (90th percentile of 4 h) for CTAS 4/5 patients [3]. For admitted patients, CAEP recommended a median of 8 h (90th percentile of 12 h). For the LAMA proportion, the proportion of patients presenting who had a disposition of LAMA were calculated. The proportion LWBS was analogously calculated.

For each ED facility, all ED presentations that started within the same date and hour (e.g. 08:00–08:59) were calculated and hourly, facility-specific means, medians, and 90th percentiles for PIA and LOS were determined. This approach condensed the multiple individual patient PIAs and LOSs per hour into representative values per hour at each facility to produce the metrics. We refer to the hourly, facility-specific averages as metrics PIA-A and LOS-A for the PIA and LOS, respectively, to allow for easier presentation. Similarly, we use PIA-M and LOS-M to represent the hourly, facility-specific medians of the PIA and LOS, respectively, and PIA-90 and LOS-90 represent the hourly, facility-specific 90th percentiles of the PIA and LOS, respectively. To form day aggregated metrics, the facility specific proportion of LAMA and proportion of LWBS were used for presentations that started on the same date.

### Data analysis

An a priori protocol and analytic plan was created. Numerical summaries (i.e., medians, IQRs represented as [25th percentile, 75th percentile]) and counts (percentages) describe patient demographics and ED presentation characteristics. Statistical analyses were conducted in R (Vienna, Austria; Version 3.5.1) [14].

## Results

**Fig. 1:**
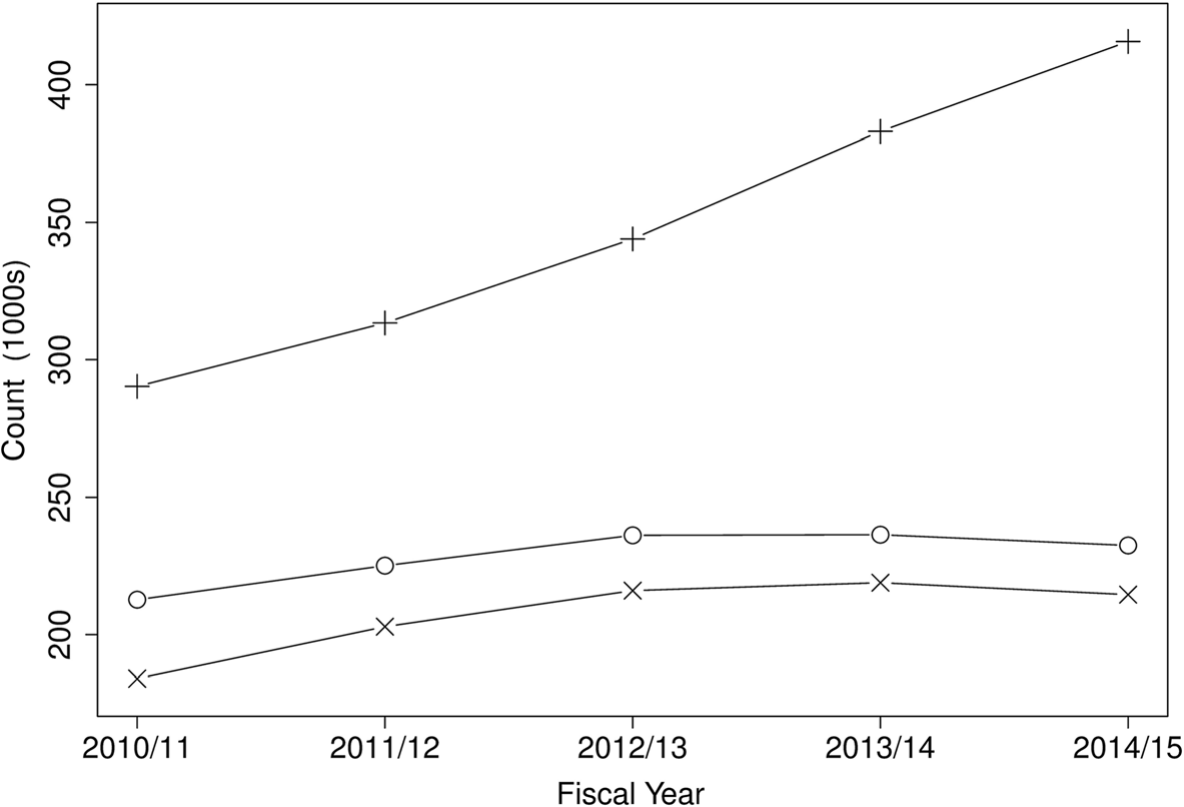
ED presentations by fiscal year and ED category: regional (○), urban (+), and academic/teaching (×)

There were 3,925,457 presentations made by 1,420,679 adults to the high volume EDs for all conditions. Table 1 provides demographic and ED presentation characteristics for the whole dataset and by ED category. The number of presentations was approximately similar for each sex (51% female) and the average age of patients was 46 years. Approximately 44% of the presentations were to urban EDs and the urban areas of Edmonton and Calgary had the most ED presentations. ED presentations increased during the study years, especially for urban EDs (Fig. 1), and there were approximately similar numbers of presentations per month, with the daytime period seeing the largest proportion of presentations. The three categories of EDs had similar distributions on most characteristics; however, academic/teaching EDs had more urgent triage scores and higher numbers of admissions than urban and regional EDs (23.0, 12.6, and 10.6% admitted in academic/teaching, urban, and regional EDs, respectively).

**Table 1:** Demographic and ED presentation characteristics for all EDs and by ED category

Characteristic	All EDs (*n* = 3,925,457)	Regional EDs (*n* = 1,142,807)	Urban EDs (*n* = 1,746,324)	Academic/ Teaching EDs (*n* = 1,036,326)
Sex, n (%)				
Female	2,020,113 (51.5)	567,661 (49.7)	937,529 (53.7)	514,923 (49.7)
Male	1,905,329 (48.5)	575,141 (50.3)	808,788 (46.3)	521,400 (50.3)
Age (years)				
mean (SD)	46.0 (19.9)	43.2 (18.9)	46.7 (20.0)	47.9 (20.4)
median [IQR]	43.0 [29, 60]	40.0 [27, 55]	44.0 [30, 61]	46.0 [30, 63]
Missing	82	0	27	55
Zone of residence, n (%)				
Z5 North	486,626 (12.4)	426,998 (37.4)	22,928 (1.3)	36,700 (3.5)
Z4 Edmonton	1,466,128 (37.3)	25,830 (2.3)	864,932 (49.5)	575,366 (55.5)
Z3 Central	272,482 (6.9)	232,799 (20.4)	15,284 (0.9)	24,399 (2.4)
Z2 Calgary	1,176,632 (30.0)	19,671 (1.7)	797,869 (45.7)	359,092 (34.7)
Z1 South	363,901 (9.3)	353,557 (30.9)	4780 (0.3)	5564 (0.5)
Missing	159,688 (4.1)	83,952 (7.3)	40,531 (2.3)	35,205 (3.4)
Fiscal year, n (%)				
2010/2011	686,976 (17.5)	212,693 (18.6)	290,250 (16.6)	184,033 (17.8)
2011/2012	741,312 (18.9)	225,100 (19.7)	313,346 (17.9)	202,866 (19.6)
2012/2013	796,151 (20.3)	236,166 (20.7)	343,978 (19.7)	216,007 (20.8)
2013/2014	838,295 (21.4)	236,380 (20.7)	383,032 (21.9)	218,883 (21.1)
2014/2015	862,723 (22.0)	232,468 (20.3)	415,718 (23.8)	214,537 (20.7)
Month of year, n (%)				
January	331,948 (8.5)	95,920 (8.4)	148,639 (8.5)	87,389 (8.4)
February	305,191 (7.8)	88,778 (7.8)	136,347 (7.8)	80,066 (7.7)
March	335,723 (8.6)	95,972 (8.4)	150,839 (8.6)	88,912 (8.6)
April	312,766 (8.0)	92,314 (8.1)	136,495 (7.8)	83,957 (8.1)
May	332,455 (8.5)	97,189 (8.5)	146,458 (8.4)	88,808 (8.6)
June	326,939 (8.3)	94,589 (8.3)	146,142 (8.4)	86,208 (8.3)
July	343,412 (8.7)	99,972 (8.7)	153,370 (8.8)	90,070 (8.7)
August	343,602 (8.8)	100,098 (8.8)	153,148 (8.8)	90,356 (8.7)
September	327,555 (8.3)	95,996 (8.4)	145,650 (8.3)	85,909 (8.3)
October	329,296 (8.4)	96,936 (8.5)	146,008 (8.4)	86,352 (8.3)
November	308,984 (7.9)	89,948 (7.9)	137,093 (7.9)	81,943 (7.9)
December	327,586 (8.3)	95,095 (8.3)	146,135 (8.4)	86,356 (8.3)
Day of week, n (%)				
Weekday (Mon-Fri)	2,803,405 (71.4)	805,493 (70.5)	1,254,413 (71.8)	743,499 (71.7)
Weekend (Sat, Sun)	1,122,052 (28.6)	337,314 (29.5)	491,911 (28.2)	292,827 (28.3)
Time of day, n (%)				
Daytime (08:01–16:00)	1,807,622 (46.0)	538,397 (47.1)	798,406 (45.7)	470,819 (45.4)
Evening (16:01–24:00)	1,461,638 (37.2)	421,100 (36.8)	657,150 (37.6)	383,388 (37.0)
Night (00:01–08:00)	656,197 (16.7)	183,310 (16.0)	290,768 (16.7)	182,119 (17.6)
Triage level, n (%)				
1 Resuscitation	29,242 (0.7)	3286 (0.3)	8974 (0.5)	16,982 (1.6)
2 Emergency	724,574 (18.5)	89,994 (7.9)	367,420 (21.0)	267,160 (25.8)
3 Urgent	1,786,753 (45.5)	439,474 (38.5)	849,733 (48.7)	497,546 (48.0)
4 Semi-urgent	1,133,670 (28.9)	513,555 (44.9)	415,238 (23.8)	204,877 (19.8)
5 Non-urgent	243,252 (6.2)	89,110 (7.8)	104,697 (6.0)	49,445 (4.8)
Missing	7966 (0.2)	7388 (0.6)	262 (0.0)	316 (0.0)
Disposition, n (%)				
Discharged	3,076,395 (78.4)	954,016 (83.5)	1,405,635 (80.5)	716,744 (69.2)
Admitted	580,325 (14.8)	120,827 (10.6)	220,852 (12.6)	238,646 (23.0)
Transferred	60,055 (1.5)	9573 (0.8)	36,490 (2.1)	13,992 (1.4)
LWBS	162,056 (4.1)	47,486 (4.2)	65,062 (3.7)	49,508 (4.8)
LAMA	41,884 (1.1)	10,047 (0.9)	16,564 (0.9)	15,273 (1.5)
Death	4742 (0.1)	858 (0.1)	1721 (0.1)	2163 (0.2)

*IQR* 25th percentile, 75th percentile, *n* count, *SD* Standard deviation

The facility-specific hourly ED crowding metrics were calculated for the 16 EDs. Fourteen facilities were operational for the full 5 years while two urban EDs started at 7 am on January 14, 2013, and May 21, 2014 (a total of 640,452 facility-specific hours during the study period). Of the 3,925,457 presentations, 3,020,762 were used in the calculation of PIA metrics (162,056 excluded because of LWBS or LAMA, 742,639 had missing time data), 3,029,451 were used in the calculation of ED LOS metrics for discharged (46,944 presentation ending in discharge had time of the disposition decision missing), and 580,280 were used in the calculation of ED metrics for admitted (45 presentations ending in admission had time the patient left the ED missing). Further, some facilities and hours had missing values because presentations with non-missing times did not meet the disposition requirements (e.g., PIA metrics based on 577,929 facility-specific hours, ED LOS metrics for discharged based on 608,797 facility-specific hours, and ED LOS for admitted based on 317,081 facility-specific hours).

**Fig. 2:**
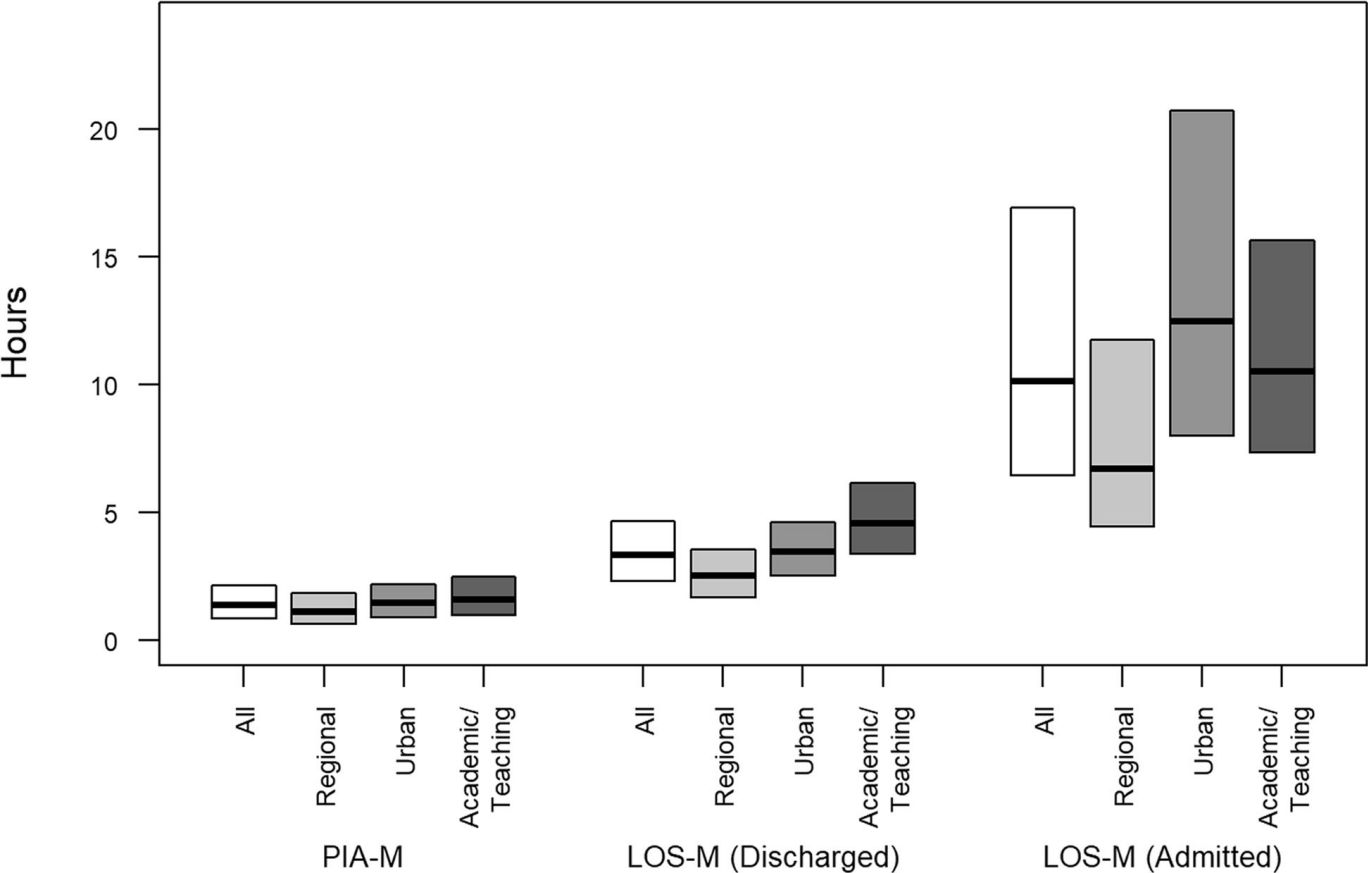
Median and interquartile range for metrics PIA-M and LOS-M for all EDs and by ED category. Legend: PIA-M = hourly, facility specific median time to physician initial assessment, LOS-M = hourly, facility specific median LOS

The median of the PIA-M metric was 1 h23m across all facilities and hours, with presentations in the urban and academic/teaching EDs having a PIA-M of 19 and 28 min higher than the regional EDs, respectively (Table 2, Fig. 2). The PIA-A times were slightly longer than the PIA-M times. The PIA-90 metric had a median of 2h13m. The PIA metrics remained relatively stable over time, although there were increases in the most recent years for academic/teaching EDs (eFigure 1).

**Table 2:** Summaries of hourly, facility-specific crowding metrics for all EDs and by ED category

Crowding Metric	All EDs	Regional EDs	Urban EDs	Academic/Teaching EDs
PIA-A				
median [IQR]	01h30m [00h57m, 02h12m]	01h11m [00h43m, 01h51m]	01h34m [01h01m,02h14m]	01h48m [01h11m, 02h33m]
PIA-M				
median [IQR]	01h23m [00h51m, 02h08m]	01h08m [00h40m, 01h50m]	01h27m [00h55m, 02h10m]	01h36m [00h59m, 02h28m]
PIA-90				
median [IQR]	02h13m [01h19m, 03h20m]	01h37m [00h55m, 02h33m]	02h18m [01h26m, 03h20m]	02h59m [01h54m, 04h17m]
LOS-A for patients discharged				
median [IQR]	03h44m [02h37m, 05h08m]	02h47m [01h57m, 03h51m]	03h53m [02h54m, 05h06m]	05h08m [03h56m, 06h44m]
LOS-M for patients discharged				
Median [IQR]	03h21m [02h18m, 04h40m]	02h31m [01h41m, 03h33m]	03h29m [02h32m, 04h38m]	04h36m [03h24m, 06h10m]
LOS-90 for patients discharged				
median [IQR]	05h26m [03h39m, 07h42m]	03h59m [02h41m, 05h36m]	05h40m [04h03m, 07h38m]	07h43m [05h46m, 10h12m]
LOSA for patients admitted				
median [IQR]	10h38m [06h40m, 17h06m]	06h45m [04h29m, 12 h07m]	12h55m [08h16m, 20h39m]	11h10m [07h49m, 16h03m]
LOSM for patients admitted				
median [IQR]	10h08m [06h28m, 16h56m]	06h42m [04h27m, 11h45m]	12h29m [08h00m, 20h43m]	10h30m [07h21m, 15h39m]
LOS-90 patients admitted				
median [IQR]	12h19m [07h17m, 21h08m]	07h12m [04h42m, 13h22m]	15h02m [09h04m, 00h06m]	13h51m [09h14m, 21h02m]
Percent LWBS (Day aggregated)				
median [IQR]	3.4 [1.7,5.7]	3.4 [1.6,5.7]	3.2 [1.6,5.4]	3.7 [1.8,6.4]
Percent LAMA (Day aggregated)				
median [IQR]	0.8 [0.0,16.]	0.6 [0.0,1.2]	0.8 [0.0,1.6]	1.1 [0.5,2.0]

*IQR* 25th percentile, 75th percentile, *LAMA* Left against medical advice, *LOS* Length of stay, *LWBS* Left without being seen, *SD* Standard deviation, *PIA-A* hourly, facility specific average time to physician initial assessment, *PIA-M* hourly, facility specific median time to physician initial assessment, *PIA-90* hourly, facility specific 90th percentile of time to physician initial assessment, *LOS-A* hourly, facility specific average LOS, *LOS-M* hourly, facility specific median LOS, *LOS-90* hourly, facility specific 90th percentile of LOS

**Fig. 3:**
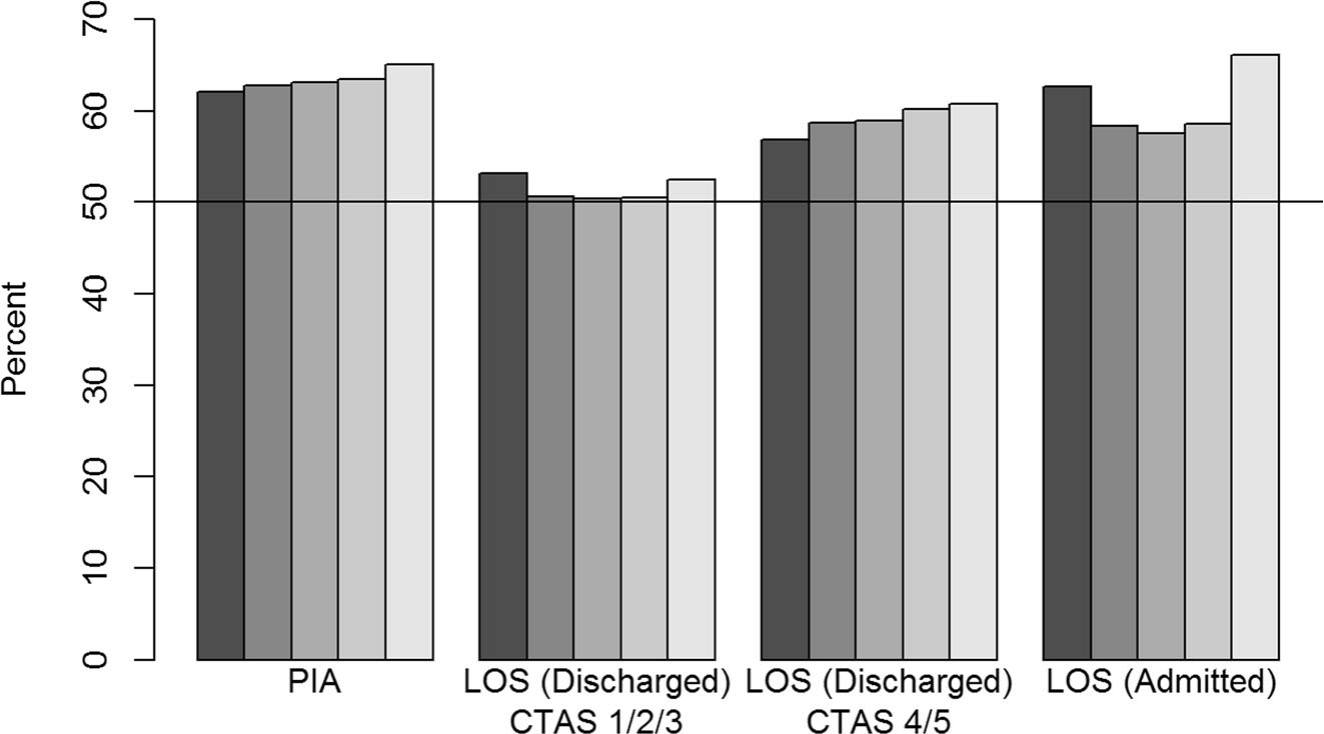
Percent of presentations from all ED that exceeded the 50th percentile CAEP recommendations. Physician initial assessment (PIA), length of stay (LOS) for discharges with CTAS 1/2/3, LOS for discharges with CTAS 4/5, and LOS for admissions for all EDs and by fiscal year (darkest grey 2010/2011, lightest grey 2014/2015). If the recommendation for the median was achieved, the bar would be below the 50% line

**Fig. 4:**
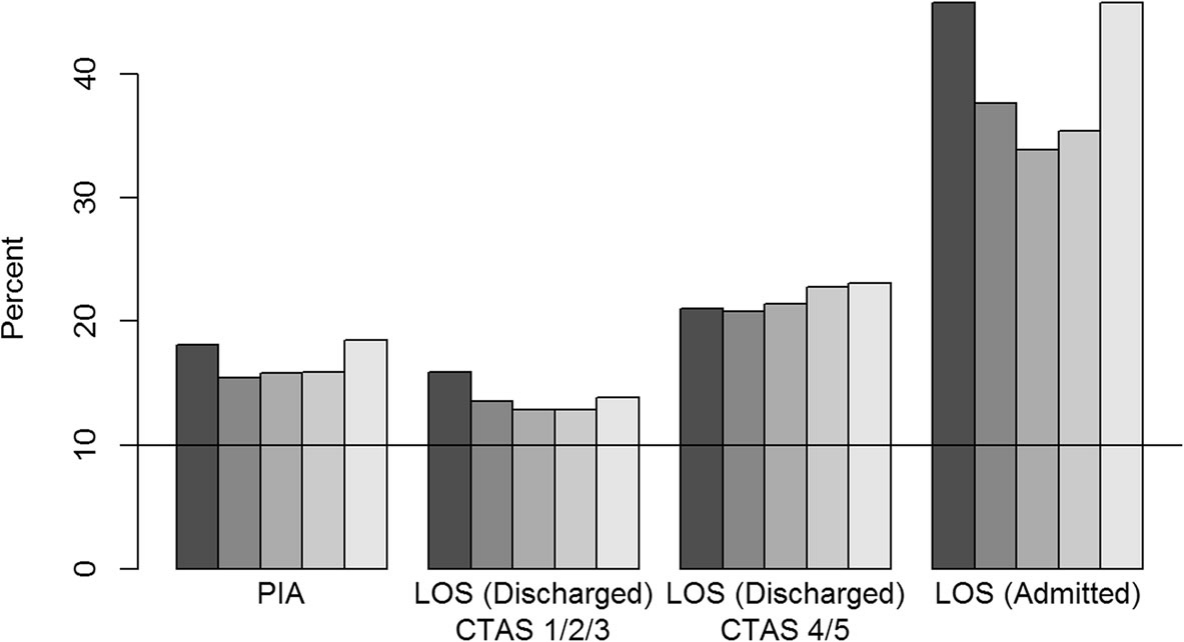
Percent of presentations from all ED that exceeded the 90th percentile CAEP recommendations. Physician initial assessment (PIA), length of stay (LOS) for discharges with CTAS 1/2/3, LOS for discharges with CTAS 4/5, and LOS for admissions for all EDs and by fiscal year (darkest grey 2010/2011, lightest grey 2014/2015). If the recommendation for the 90th percentile was achieved, the bar would be below the 10% line

When compared against the CAEP recommendations of 1 h for the median and 3 h for the 90th percentile, PIAs for individual presentations exceeded the recommendations: 63.3% (1,912,545/3020762) of presentations had PIA longer than 1 hour and 16.8% (506613) were longer than 3 hours (Figs. 3 and 4, eTable 1 in the Supplementary file). Urban and academic/teaching EDs exceeded the recommendations more frequently and later years saw increases for the academic/teaching EDs (eFigures 2 and 3).

For presentations that ended in discharge, the median LOS-M metric was 3h21m. The regional EDs had the shortest median LOS-M (2h31m), whereas the urban and academic/teaching EDs had longer medians for LOS-Ms, 3h29m and 4h36m (Fig. 2), respectively. The medians of the LOS-As were approximately 20 m longer than the medians of the LOS-Ms. The median of the LOS-90 metric for discharges was approximately 5.5 h for all EDs combined (5h26m) with the greatest values in the academic/teaching EDs (median = 7h43m). The metrics remained relatively stable over time (eFigure 4).

For the CTAS 1/2/3 levels, the presentations that ended in discharge had LOSs that were close to the median recommendation of 4 h by CAEP (51.4%, 937,497/1824489) but larger than the 90th percentile recommendation of 8 h (13.7%, 250,456). The performance against CAEP benchmarks remained relatively stable over time (eTable 2, Figs. 3 and 4). Regional EDs tended to meet the recommendations whereas urban and academic/teaching EDs were least likely to meet the recommendations, especially in the more recent years (eFigure 2). For the CTAS 4/5 patients who were discharged, LOS exceeded the CAEP recommendation for a median LOS of 2 h (59.2%, 709,304/1199053 exceeding 2 h) and 90th percentile recommendation of 4 h (21.9%, 262,427 exceeding 4 h). Failure to meet CAEP recommendations for CTAS 4/5 patients increased in frequency over the years (Figs. 3 and 4). Almost all years and all ED categories exceeded the recommendations with the urban and academic/teaching EDs meeting the recommendations less frequently in later years (eFigures 2 and 3).

The LOS for presentations ending in admission were considerably longer than those ending in discharge. The median LOS-M for all EDs together was approximately 10 h (10h08m, Fig. 2). Regional EDs had the lowest median LOS-M (6h42m) whereas the urban EDs had a value of 12 h29m and academic/teaching EDs had a value of 10h30m. The urban EDs had the largest values of the ED categories for LOS admissions metrics based on the other LOS metrics as well. The metrics varied over time with urban EDs showing the most variability and regional EDs increasing over the years (eFigure 5).

Among admitted patients, 60.7% (351,986/580280) had ED LOS longer than the CAEP recommended median LOS of 8 h, and 39.7% (230101) had LOS longer than the CAEP-recommended 90th percentile of 12 h. Initially, the LOSs were becoming closer to the recommendations and then increased in later years (eTable 1, Figs. 3 and 4). The urban and academic/teaching EDs exceeded the recommendations each year with an irregular pattern whilst the regional EDs have increased in the 90th percentile recommendation over time (eFigures 2 and 3).

Relatively few ED presentations had a disposition that was classified as LWBS (162,056, 4.1% of all ED presentations) and even less for LAMA (41,884, 1.1% of all ED presentations). The median percentage of LWBS was 3.4% in the regional EDs, 3.7% for academic/teaching EDs, and 3.2% for urban EDs (Table 2). LAMA was about 1% (eFigure 6) for each ED category, with 1.1% for academic/teaching EDs. These values have varied over time for the different ED categories (eFigures 7 and 8).

## Discussion

This large population-based study described facility-specific hourly crowding metrics that have been considered indicative of ED crowding [3] and that could be calculated from reliable and valid administrative data on presentations by adults. The study focused on high volume EDs where crowding may be more likely to occur and classified these EDs into three categories: regional, urban, and academic/teaching. Demographics of patients presenting and trends in timing of presentation were similar across ED categories; however, the urban and academic/teaching EDs received patients who required more urgent care, spent longer in the ED and more often required hospitalization.

The first important finding is that ED volumes continue to increase at all sites. The reasons for this observation are multi-factorial. While the annual Alberta population increased from 3.79 to 4.20 million over the study period and the number of ED presentations also increased (1.94 to 2.18 million), the crude rates were relatively stable at 512.4 to 519.8 ED presentations per 1000 population, suggesting that population growth during the study period contributed to ED volume increases [15, 16]. The proportion of adult patients without a primary care provider (PCP) has hovered at approximately 20% in the province, and an even higher proportion of ED patients have been shown to be disconnected from primary care [17]. Moreover, standard scheduling practices, access to medical/health information, a provincial HealthLink line for advice and other factors coalesce to make the ED a preferred option for many patients [17].

During our study period, the CAEP recommendations were frequently exceeded, with urban and academic/teaching EDs more likely to exceed the recommendations. For PIA, 63% of the presentations were longer than the recommended median of 1 h. For presentations ending in discharge, presentations in the CTAS 1/2/3 levels were close the median recommendation for LOS; however, the CTAS 4/5 levels had larger LOS than recommendations. For presentation ending in admission, LOSs frequently exceeded recommendations. The pattern of increasing crowding in recent years, with times exceeding the recommendations in the urban and academic/teaching EDs is a concern.

We observed important differences in crowding metrics between ED type. While PIA times were similar across ED types, the LOS for both discharged and admitted patients were higher in urban EDs and higher still in academic EDs. This observation may be related to the acuity and complexity of cases seen in academic centres, as witnessed by the higher CTAS scores at presentation, older age and the higher admission percentages. These observed differences also suggest that there are unique operational factors leading to ED crowding that differ among ED type that need to be addressed using site-specific strategies. For example, in academic EDs, consultation and admission processes that rely on hierarchical training team structures may disproportionately contribute to delays in ED and inpatient disposition decisions. Changing admission decision-making processes may be a strategy specific to academic EDs that can improve patient throughput and output. Finally, each ED and region has made efforts to reduce ED overcrowding, and the fidelity, effectiveness of these interventions may have influenced these results.

Finally, examples of the consequences of ED crowding are being experienced across the province. In higher volume EDs, with higher acuity, and higher admission percentages, patients are more likely to leave prior to the completion of care. For example, LWBS patients is highest in academic/teaching facilities where PIA is also highest; these proportions “improve” in regional EDs and are best at urban EDs; however, they are never acceptable. In adults, LWBS is often association with frustration and prolonged delays [18]. One could argue that LAMA is also partially related to these issues; however, mental health and addiction (MHA) issues tend to play a more important role. This is crucial, since MHA services are similarly under strain, and over-taxed MHA services may contribute disproportionally to ED crowding.

The strengths of our study include a large sample size from population-based data sources in a geographically large area of Canada. In addition, ED charts are coded by medical record nosologists who are experts in using International Classification of Diseases (ICD) diagnostic codes. The study has several limitations. First, the coders rely on paper-based charts for coding, which are notoriously incomplete and difficult to read in EDs. Second, our results may not be generalizable to other areas of Canada or other jurisdictions with different health care systems. The ED crowding metrics were not tested against important patient outcomes (e.g., in-patient LOS, re-visits to ED, death), and we cannot conclude any consequences regarding the degree of overcrowding observed. There are, however, many examples of the negative consequences associated with ED overcrowding, and it would be hard to imagine these not occurring here. Finally, there may be errors in the documented times and some times were not available; however, broadly speaking, these errors are random and likely do not influence our results.

## Conclusion

Presentations to these urban and regional EDs in Alberta are increasing over time and out of proportion to population growth. Considerable variations in important metrics of ED crowding were observed across three ED categories, which further indicate that academic/teaching sites are under particular strain. Crowding in high-volume EDs in this province frequently exceeds national recommendations, and urgent actions are required to avoid the adverse consequences that portend overcrowding.

## Supplementary information

**Additional file 1: eTable S1.** Percentages of presentations that exceed recommendations for all years and by fiscal year. **eTable S2.** Percentages of presentations that exceed recommendations by ED category for all years and by fiscal year. **eFigure S1.** Median and interquartile range (25th percentile, 75th percentile) hourly, facility-specific median physician initial assessment (PIA) times by years and by ED category. **eFigure S2.** Percent of presentations from all ED that exceeded the CAEP recommendations for medians for physician initial assessment (PIA), length of stay (LOS) for discharges with CTAS 1/2/3, LOS for discharges with CTAS 4/5, and LOS for admissions by ED category and by fiscal year (darkest grey 2010/2011, lightest grey 2014/2015). **eFigure S3.** Percent of presentations from all ED that exceeded the CAEP recommendations for 90th percentile for physician initial assessment (PIA), length of stay (LOS) for discharges with CTAS 1/2/3, LOS for discharges with CTAS 4/5, and LOS for admissions by ED category and by fiscal year (darkest grey 2010/2011, lightest grey 2014/2015). **eFigure S4.** Median and interquartile range (25th percentile, 75th percentile) for hourly, facility-specific median length of stay (LOS) for discharges by years and by ED category. **eFigure S5.** Median and interquartile range (25th percentile, 75th percentile) for hourly, facility-specific median length of stay (LOS) for admissions by years and by ED category. **eFigure S6.** Median and interquartile range (25th percentile, 75th percentile) of daily, facility-specific percent left without being seen (LWBS) and left against medical advice (LAMA) for all EDs and by ED category. **eFigure S7.** Median and interquartile range (25th percentile, 75th percentile) for daily, facility-specific percent left without being seen (LWBS) by years and by ED category. **eFigure S8.** Median and interquartile range (25th percentile, 75th percentile) for daily, facility-specific percent left against medical advice (LAMA) by years and by ED category.

## Data Availability

Data is the property of Alberta Health and the authors are not allowed to provide the data. Requests can be made for the same data from Alberta Health for researchers who meet the criteria for access to confidential data. Researchers are welcome to inquire for further information at Health.RESDATA@gov.ab.ca.

## Abbreviations

25%ile: 25th percentile
75%ile: 75th percentile
AHCIP: Alberta Health Care Insurance Plan
CAEP: Canadian Association of Emergency Physicians
CTAS: Canadian Triage and Acuity Score
ED: Emergency department
h: hours
ICD: International Classification of Diseases
ICD-10-CA: International Classification of Diseases and Related Health Problems, Tenth Revision, The Canadian Enhancement of ICD-10
IQR: Interquartile range
LAMA: Left against medical advice
LOS: Emergency department length of stay
LWBS: Left without being seen
m: minutes
n: count
NACRS: National Ambulatory Care Reporting System
PIA: Time to physician initial assessment
SD: Standard deviation
