# Supplementary material for: Temporal trends in emergency department volumes and crowding metrics in a western Canadian province: a population-based, administrative data study
Source: BMC Health Serv Res. 2020 Apr 26;20:356. doi: 10.1186/s12913-020-05196-4 (PMC7183635; doi:10.1186/s12913-020-05196-4)

Supplementary tables:

**eTable S1.** Percentages of presentations that exceed recommendations for all years and by fiscal year.

| **Metric** | **CAEP**  **Recommendation** | **All Years** | **2010/11** | **2011/12** | **2012/13** | **2013/14** | **2014/15** |
| --- | --- | --- | --- | --- | --- | --- | --- |
| PIA | > 1h : 50% | 63.3% | 62.0% | 62.7% | 63.1% | 63.4% | 65.0% |
|  | > 3h : 10% | 16.8% | 18.1% | 15.5% | 15.8% | 15.9% | 18.5% |
| LOS Discharged  (CTAS 1/2/3) | > 4h : 50% | 51.4% | 53.2% | 50.6% | 50.4% | 50.5% | 52.4% |
|  | > 8h : 10% | 13.7% | 15.9% | 13.6% | 12.9% | 12.8% | 13.9% |
| LOS Discharged  (CTAS 4/5) | > 2h : 50% | 59.2% | 56.9% | 58.7% | 58.9% | 60.1% | 60.7% |
|  | > 4h : 10% | 21.9% | 21.0% | 20.8% | 21.4% | 22.8% | 23.1% |
| LOS Admitted | > 8h : 50% | 60.7% | 62.6% | 58.3% | 57.5% | 58.6% | 66.0% |
|  | > 12h : 10% | 39.7% | 45.8% | 37.6% | 33.9% | 35.5% | 45.8% |

CAEP=Canadian Association of Emergency Physicians; LOS=length of stay; PIA=physician initial assessment

**eTable S2.** Percentages of presentations that exceed recommendations by ED category for all years and by fiscal year.

| **Metric** | **CAEP**  **Recommendation** | **Category** | **All Years** | **2010/11** | **2011/12** | **2012/13** | **2013/14** | **2014/15** |
| --- | --- | --- | --- | --- | --- | --- | --- | --- |
| PIA | > 1h : 50% | Regional | 54.9% | 50.8% | 56.9% | 57.2% | 55.6% | 54.1% |
|  |  | Urban | 65.6% | 65.7% | 65.7% | 66.0% | 64.5% | 66.3% |
|  |  | Academic/teaching | 65.5% | 64.6% | 62.0% | 62.5% | 66.9% | 71.5% |
|  | > 3h : 10% | Regional | 9.3% | 7.8% | 9.7% | 10.3% | 9.3% | 9.3% |
|  |  | Urban | 16.1% | 18.7% | 15.5% | 16.2% | 14.3% | 16.2% |
|  |  | Academic/teaching | 23.3% | 24.8% | 19.2% | 18.9% | 23.4% | 30.6% |
| LOS Discharged  (CTAS 1/2/3) | > 4h : 50% | Regional | 37.5% | 35.2% | 37.5% | 37.5% | 37.9% | 39.0% |
|  |  | Urban | 50.5% | 53.8% | 50.3% | 49.6% | 48.6% | 51.0% |
|  |  | Academic/teaching | 63.4% | 64.7% | 60.3% | 61.4% | 64.2% | 66.9% |
|  | > 8h : 10% | Regional | 7.2% | 7.2% | 7.5% | 6.7% | 7.0% | 7.7% |
|  |  | Urban | 11.9% | 14.4% | 12.3% | 11.4% | 10.5% | 11.3% |
|  |  | Academic/teaching | 22.1% | 24.4% | 20.0% | 20.0% | 21.9% | 24.8% |
| LOS Discharged  (CTAS 4/5) | > 2h : 50% | Regional | 50% | 47.3% | 52.3% | 50.6% | 49.3% | 50.5% |
|  |  | Urban | 62.4% | 61.8% | 61.1% | 62.4% | 62.7% | 63.2% |
|  |  | Academic/teaching | 74.4% | 75.2% | 71.0% | 71.1% | 76.4% | 78.4% |
|  | > 4h : 10% | Regional | 14.1% | 13.1% | 15.2% | 14.2% | 13.5% | 14.6% |
|  |  | Urban | 22.8% | 23.6% | 21.7% | 22.7% | 23.1% | 22.8% |
|  |  | Academic/teaching | 39.0% | 39.2% | 34.3% | 35.6% | 41.1% | 44.7% |
| LOS Admitted | > 8h : 50% | Regional | 39.6% | 31.1% | 34.2% | 39.8% | 43.9% | 48.0% |
|  |  | Urban | 69.4% | 72.8% | 67.2% | 66.4% | 65.4% | 75.1% |
|  |  | Academic/teaching | 63.2% | 70.1% | 62.8% | 58.4% | 59.4% | 66.1% |
|  | > 12h : 10% | Regional | 24.4% | 18.1% | 21.2% | 24.2% | 27.8% | 29.8% |
|  |  | Urban | 47.9% | 55.3% | 44.4% | 41.7% | 41.7% | 56.6% |
|  |  | Academic/teaching | 39.7% | 51.9% | 40.1% | 31.8% | 33.3% | 43.0% |

CAEP=Canadian Association of Emergency Physicians; LOS=length of stay; PIA=physician initial assessment

Supplementary figures:

**eFigure S1.** Median and interquartile range (25th percentile, 75th percentile) hourly, facility-specific median physician initial assessment (PIA) times by years and by ED category.


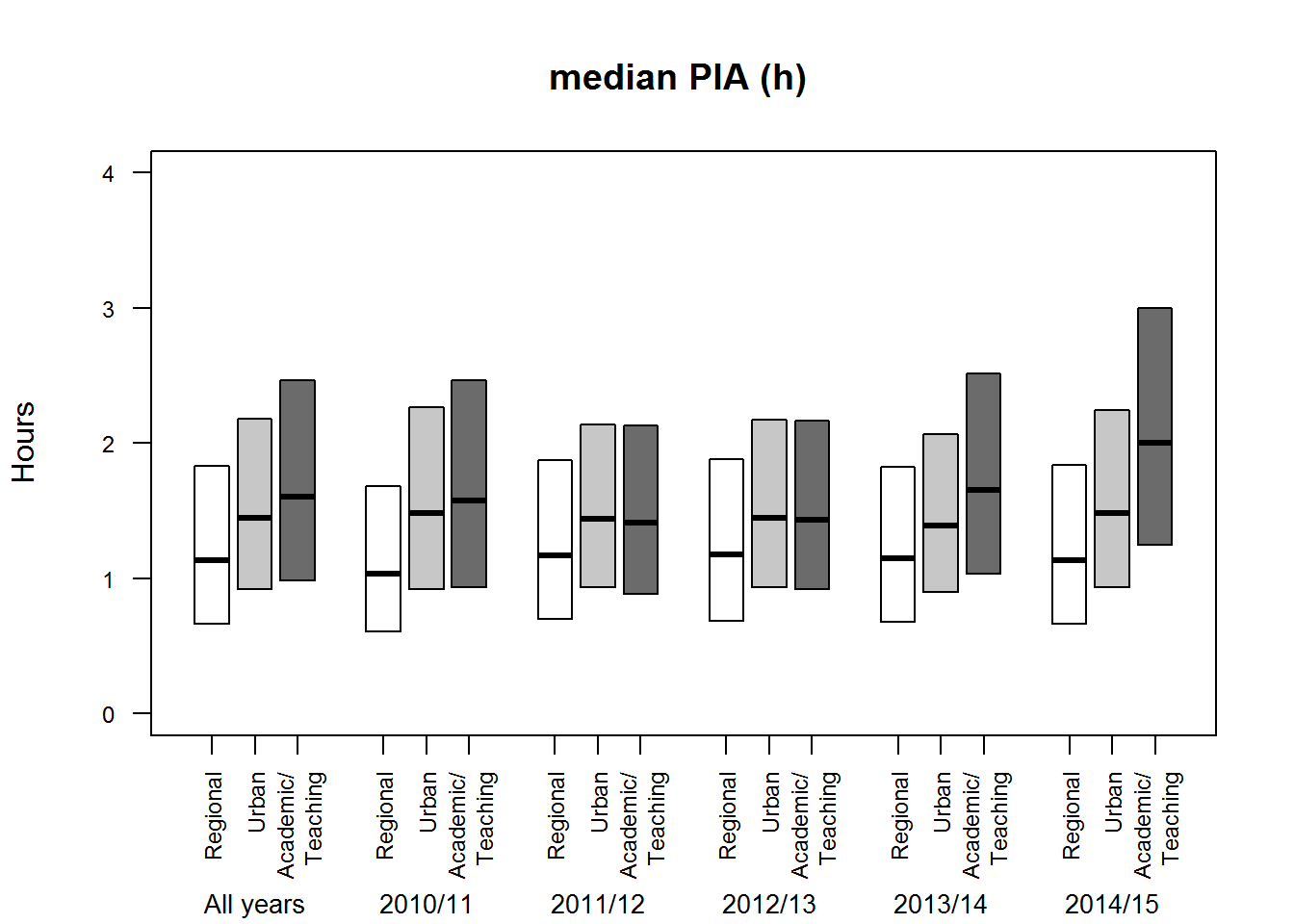


**eFigure S2.** Percent of presentations from all ED that exceeded the CAEP recommendations for medians for physician initial assessment (PIA), length of stay (LOS) for discharges with CTAS 1/2/3, LOS for discharges with CTAS 4/5, and LOS for admissions by ED category and by fiscal year (darkest grey 2010/2011,…,lightest grey 2014/2015).

| (a) PIA | (b) LOS Admitted |
| --- | --- |
| 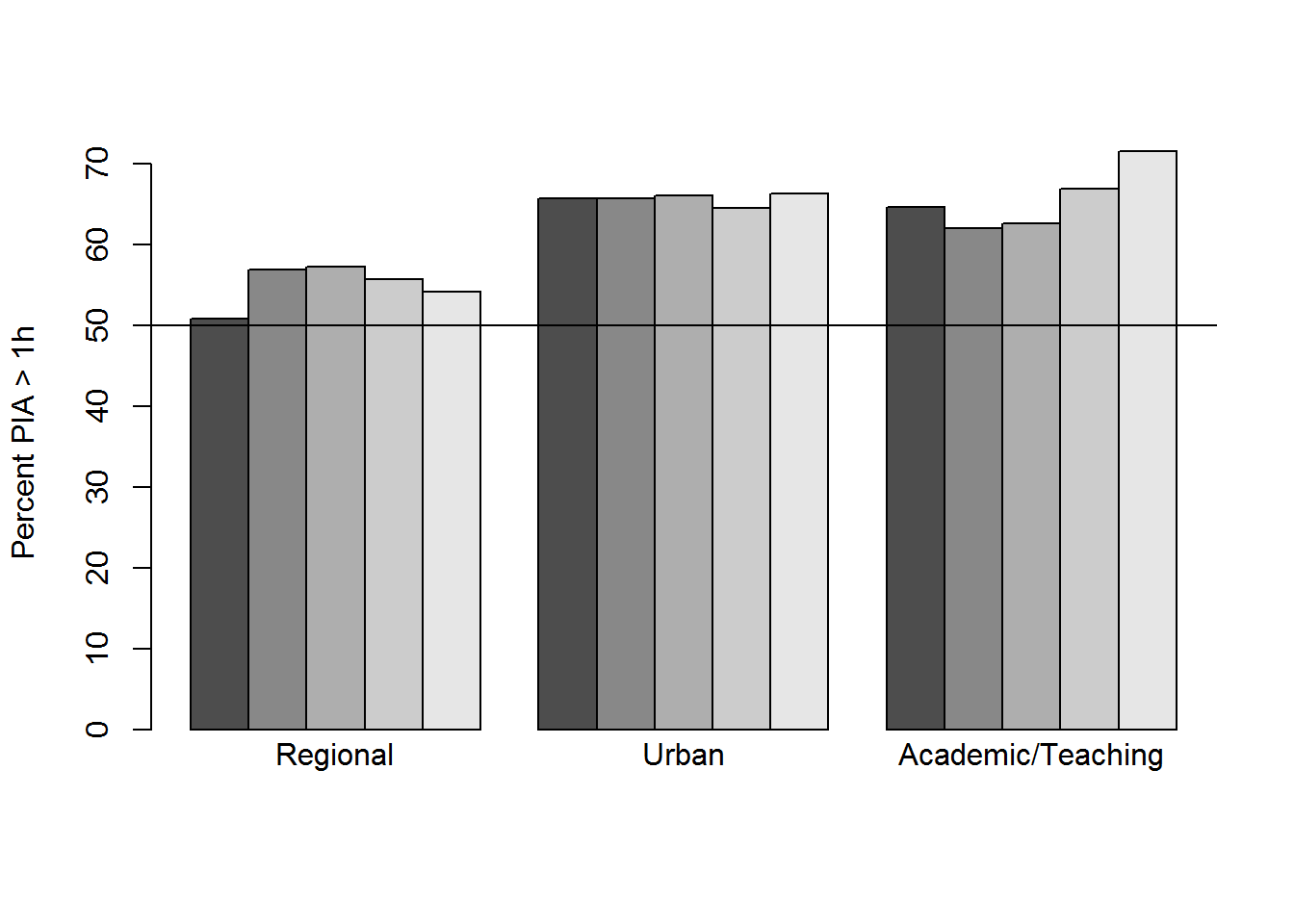 | 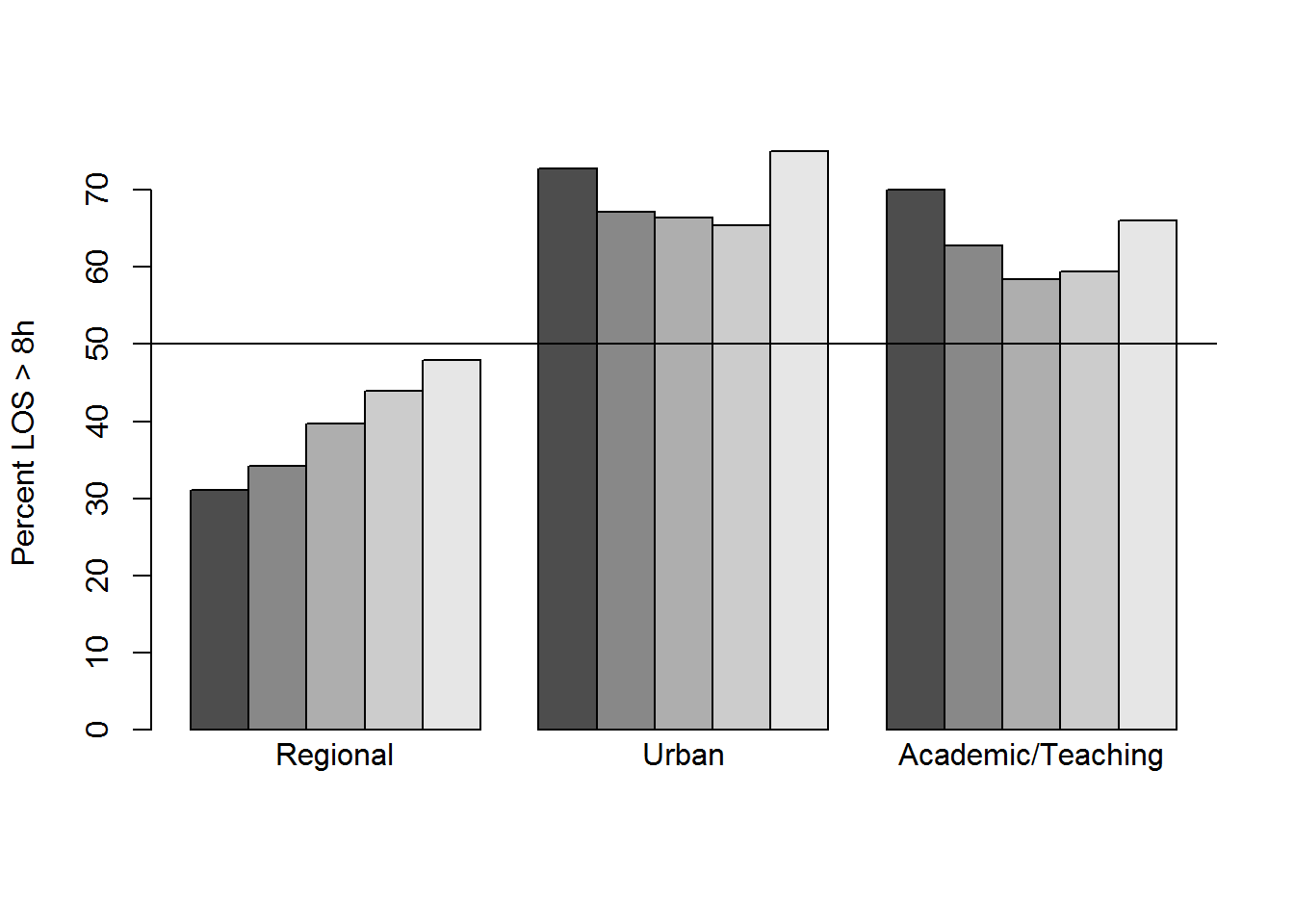 |
| (c) LOS Discharged (CTAS 1/2/3) | (d) LOS Discharged (CTAS 4/5) |
| 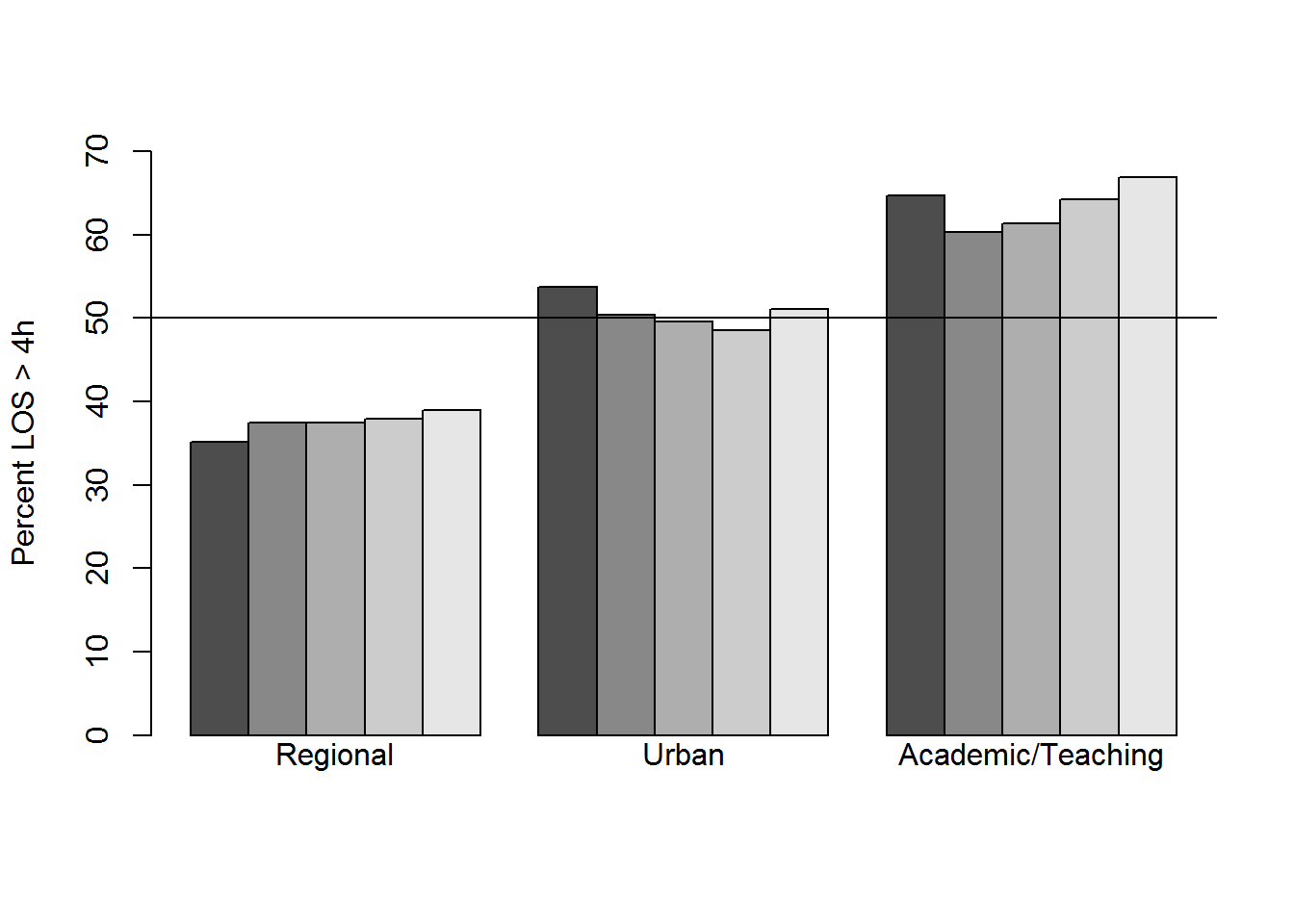 | 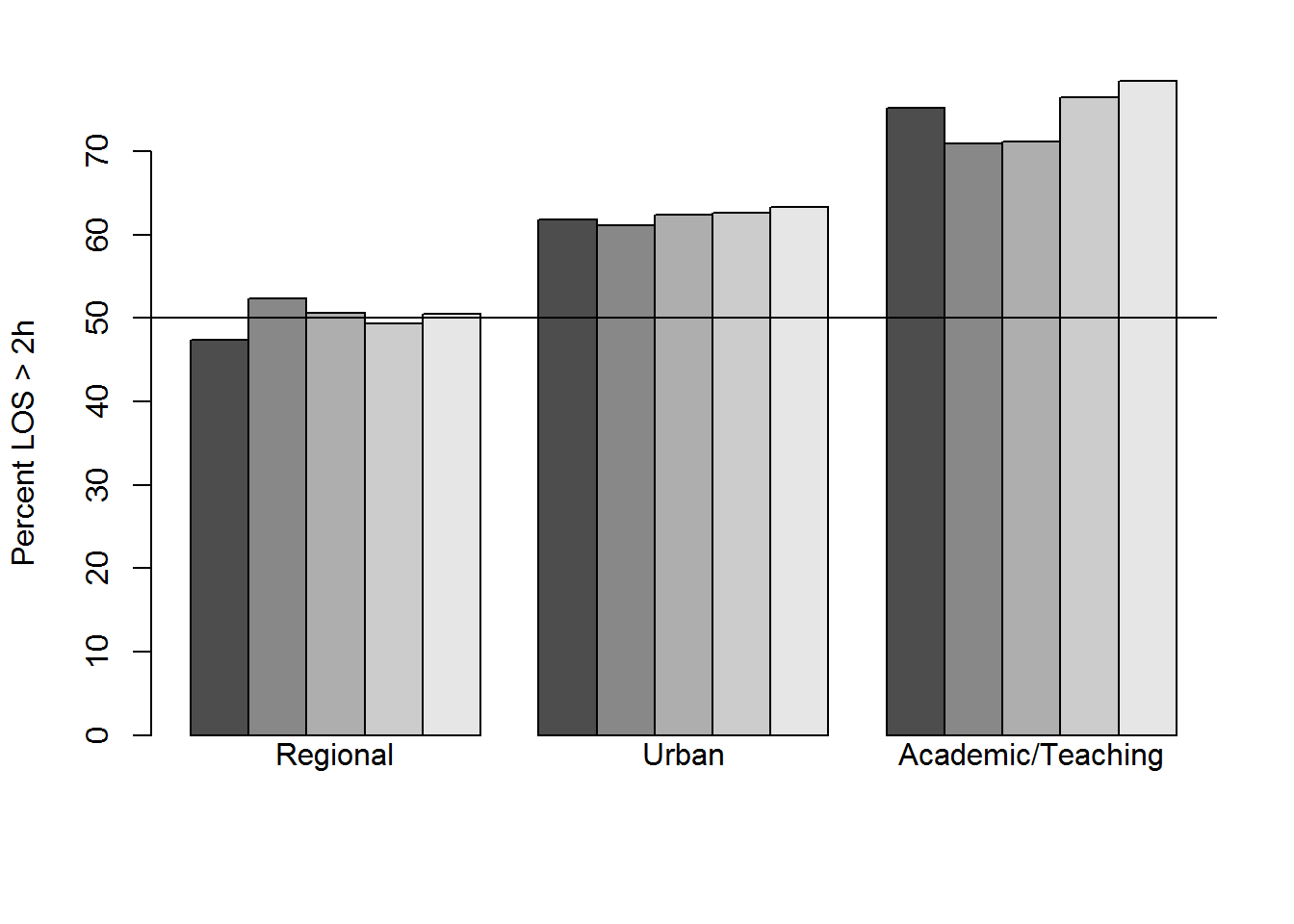 |

**eFigure S3.** Percent of presentations from all ED that exceeded the CAEP recommendations for 90^th^ percentile for physician initial assessment (PIA), length of stay (LOS) for discharges with CTAS 1/2/3, LOS for discharges with CTAS 4/5, and LOS for admissions by ED category and by fiscal year (darkest grey 2010/2011,…,lightest grey 2014/2015).

| (a) PIA | (b) LOS Admitted |
| --- | --- |
| 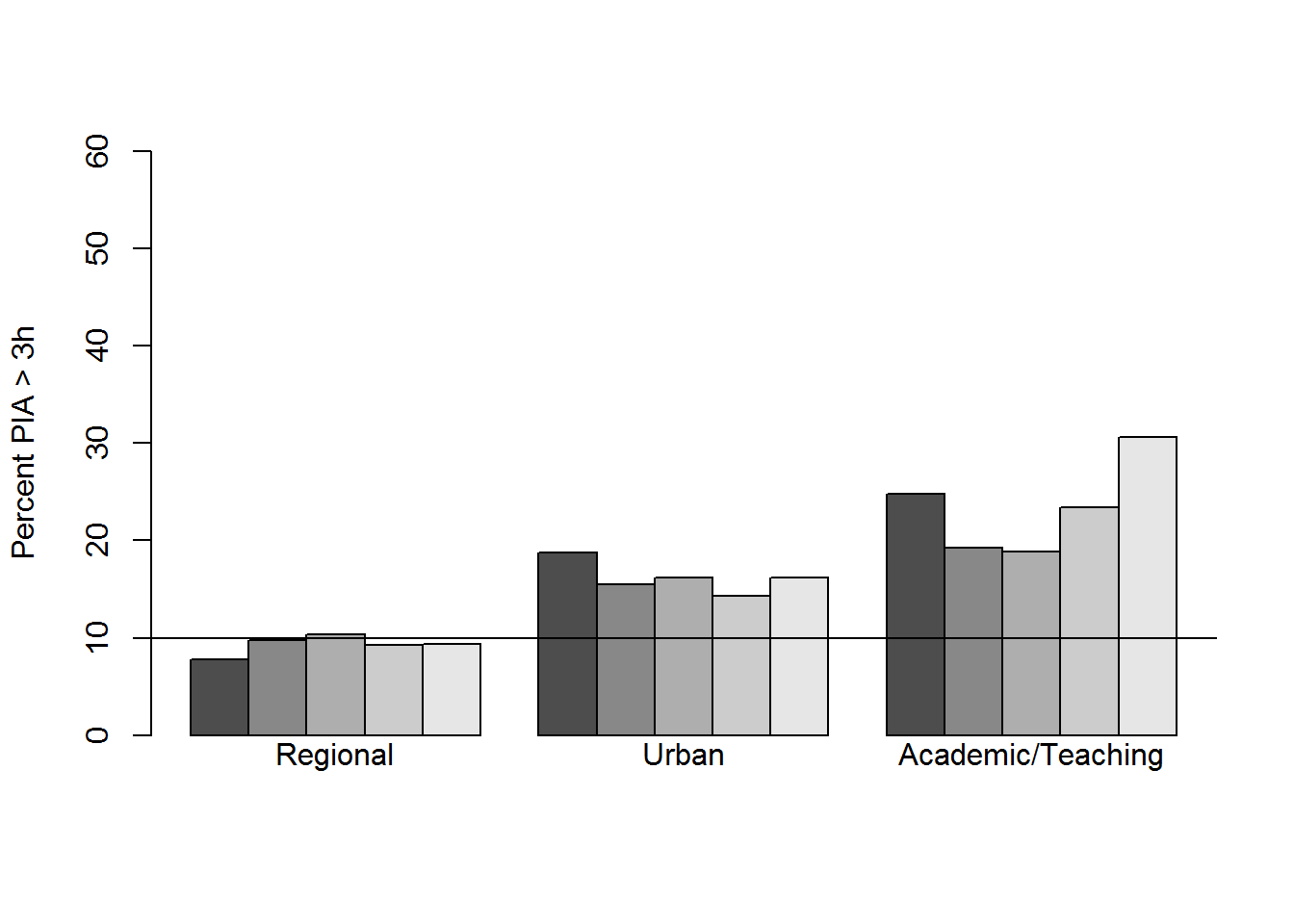 | 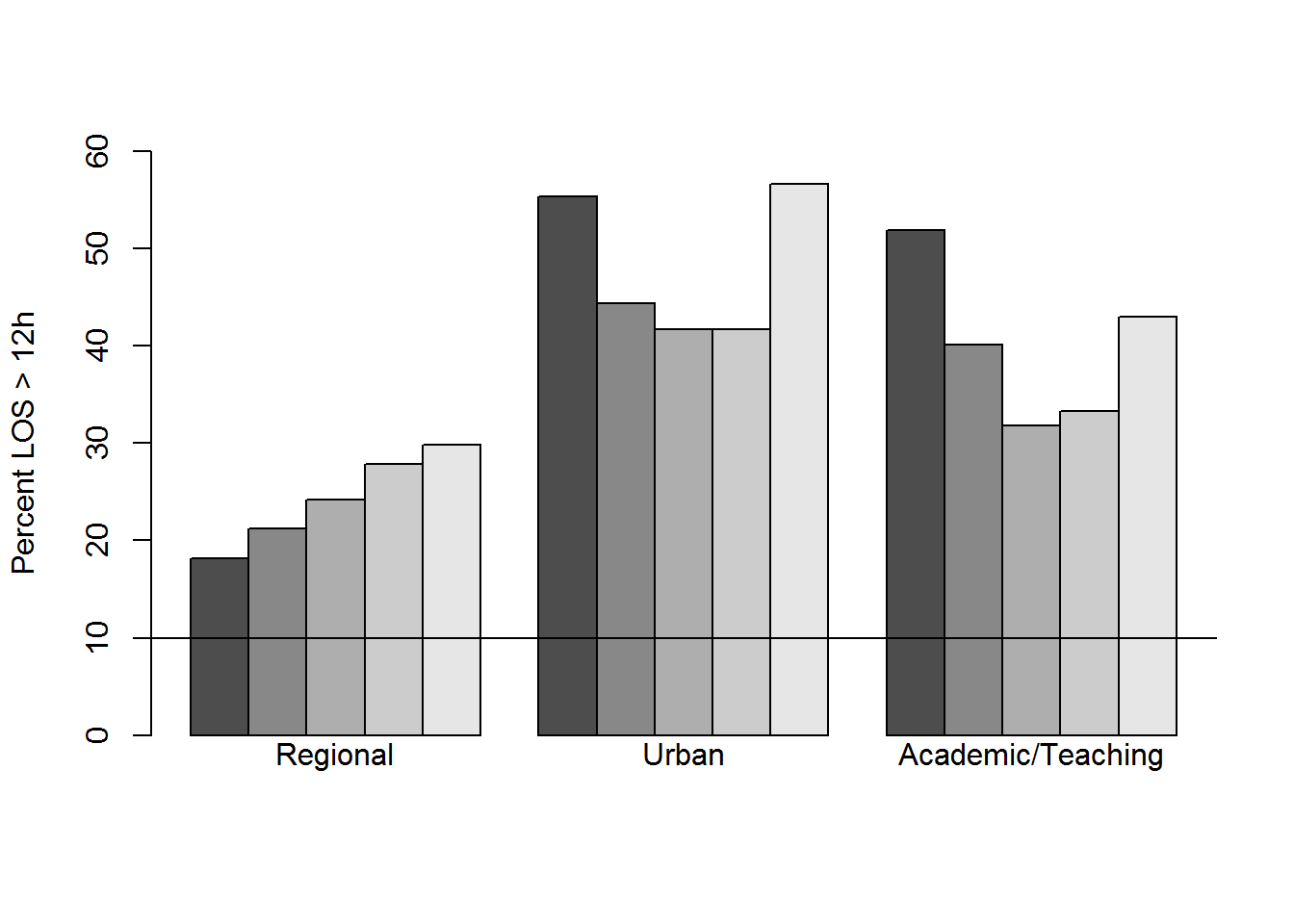 |
| (c) LOS Discharged (CTAS 1/2/3) | (d) LOS Discharged (CTAS 4/5) |
| 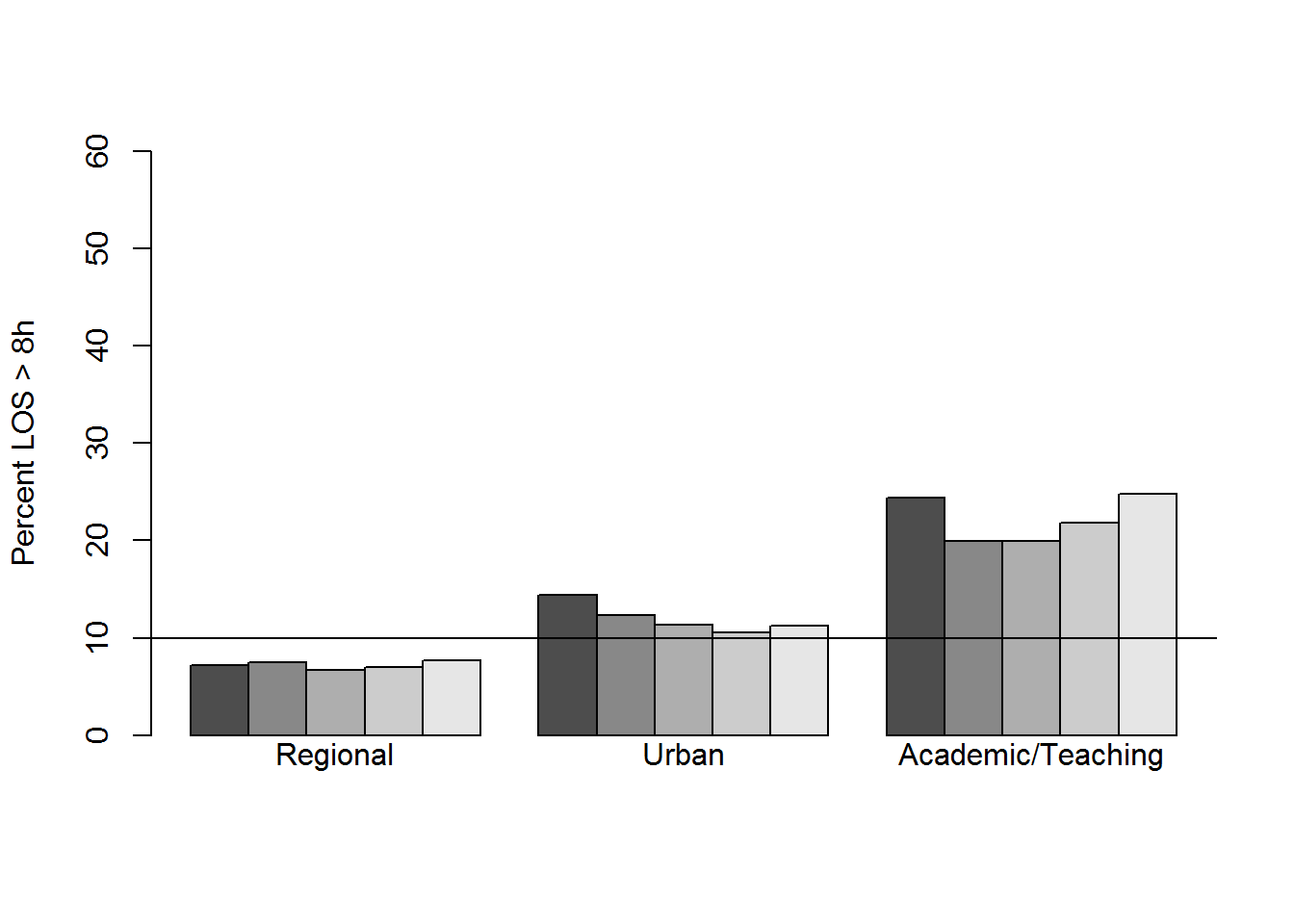 | 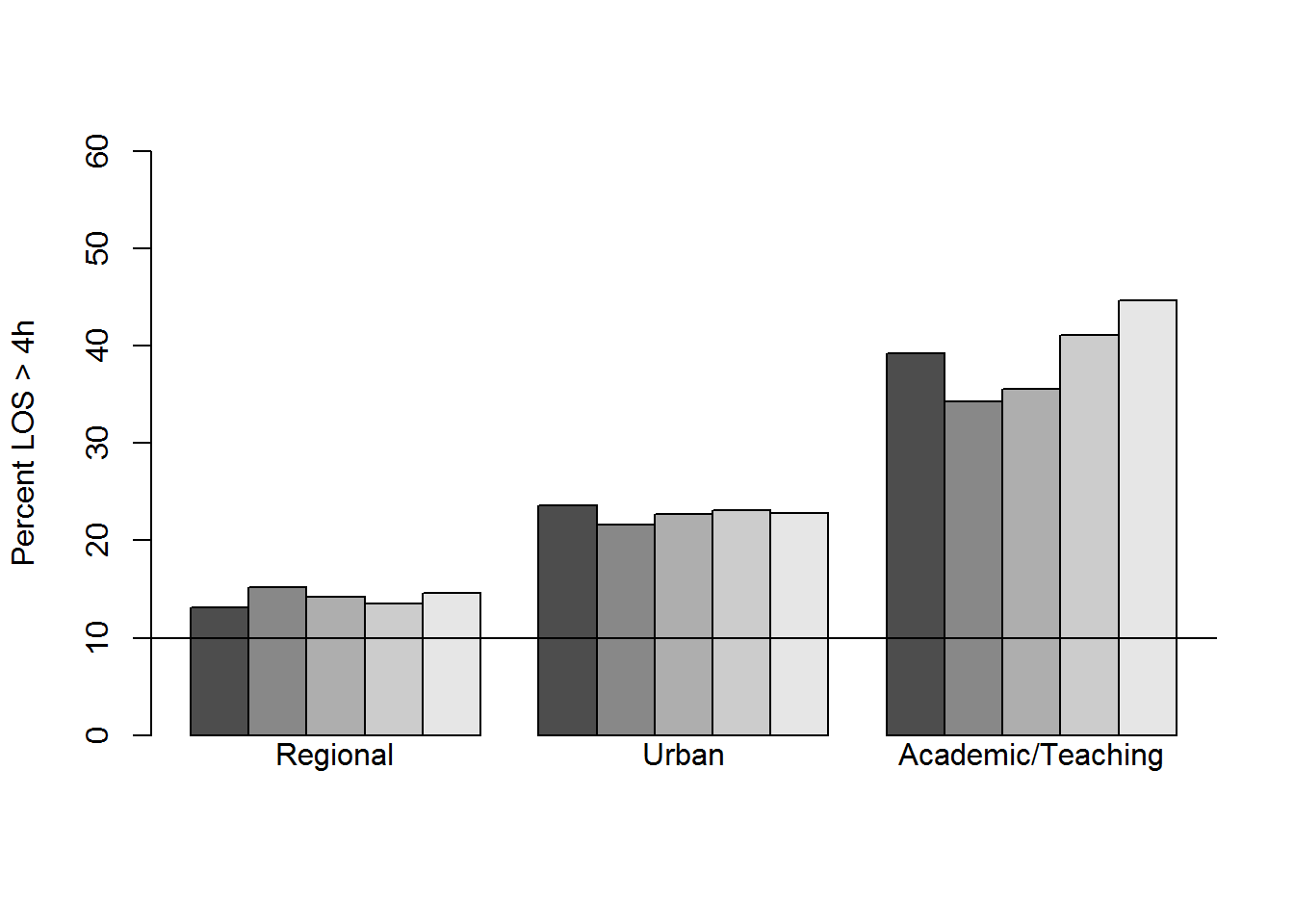 |

**eFigure S4.** Median and interquartile range (25th percentile, 75th percentile) for hourly, facility-specific median length of stay (LOS) for discharges by years and by ED category.


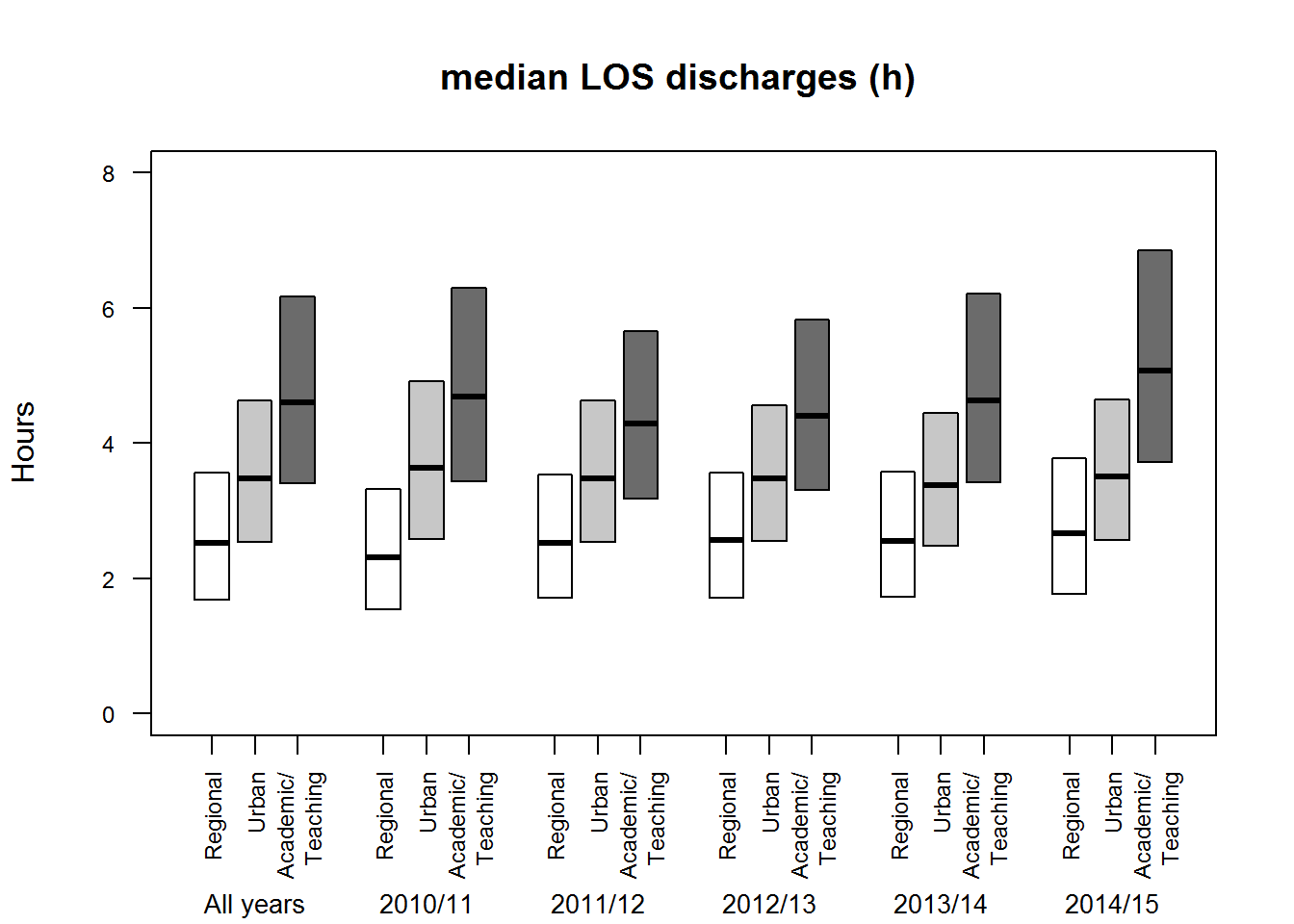


**eFigure S5.** Median and interquartile range (25th percentile, 75th percentile) for hourly, facility-specific median length of stay (LOS) for admissions by years and by ED category.


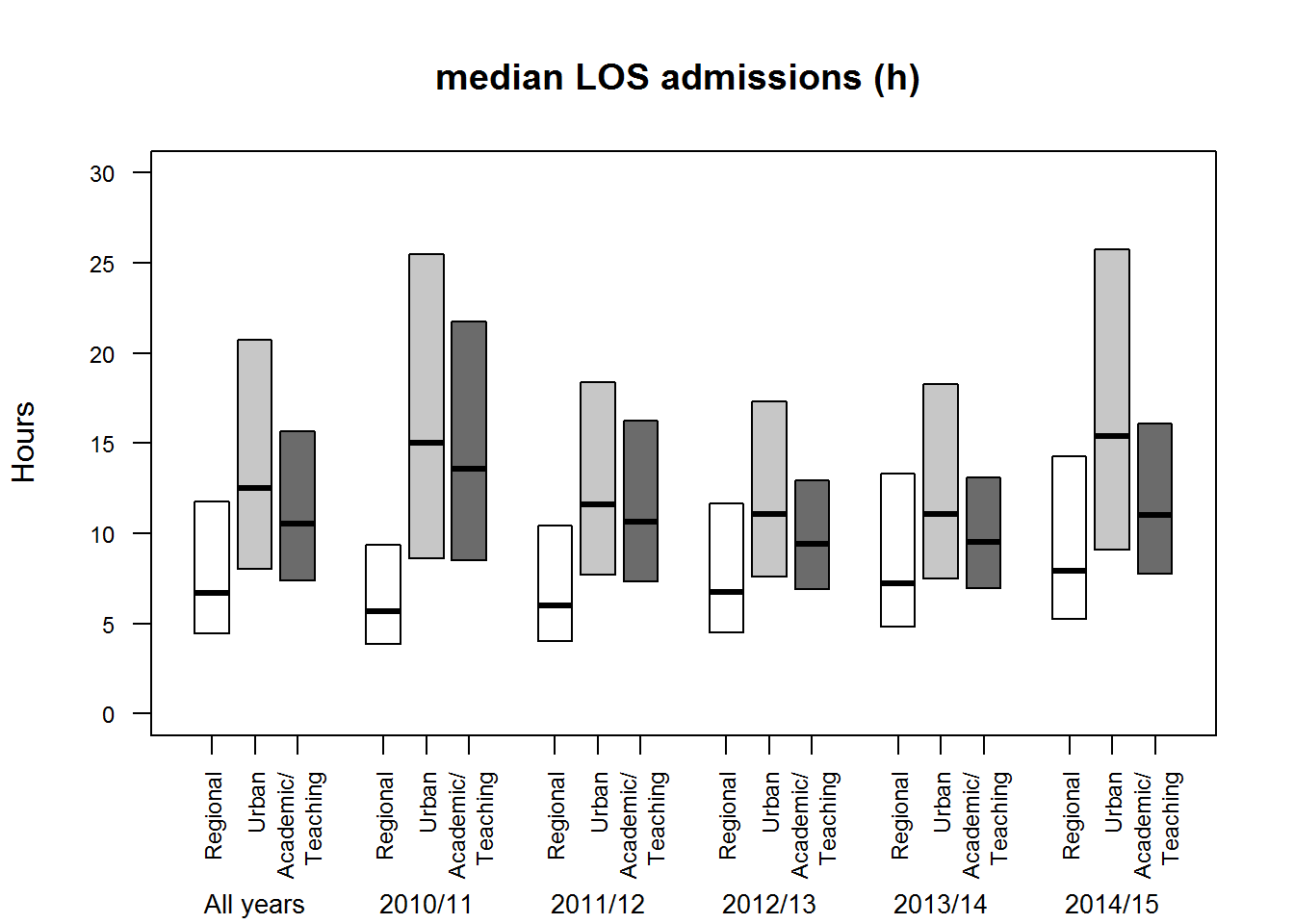


**eFigure S6.** Median and interquartile range (25^th^ percentile, 75^th^ percentile) of daily, facility-specific percent left without being seen (LWBS) and left against medical advice (LAMA) for all EDs and by ED category.


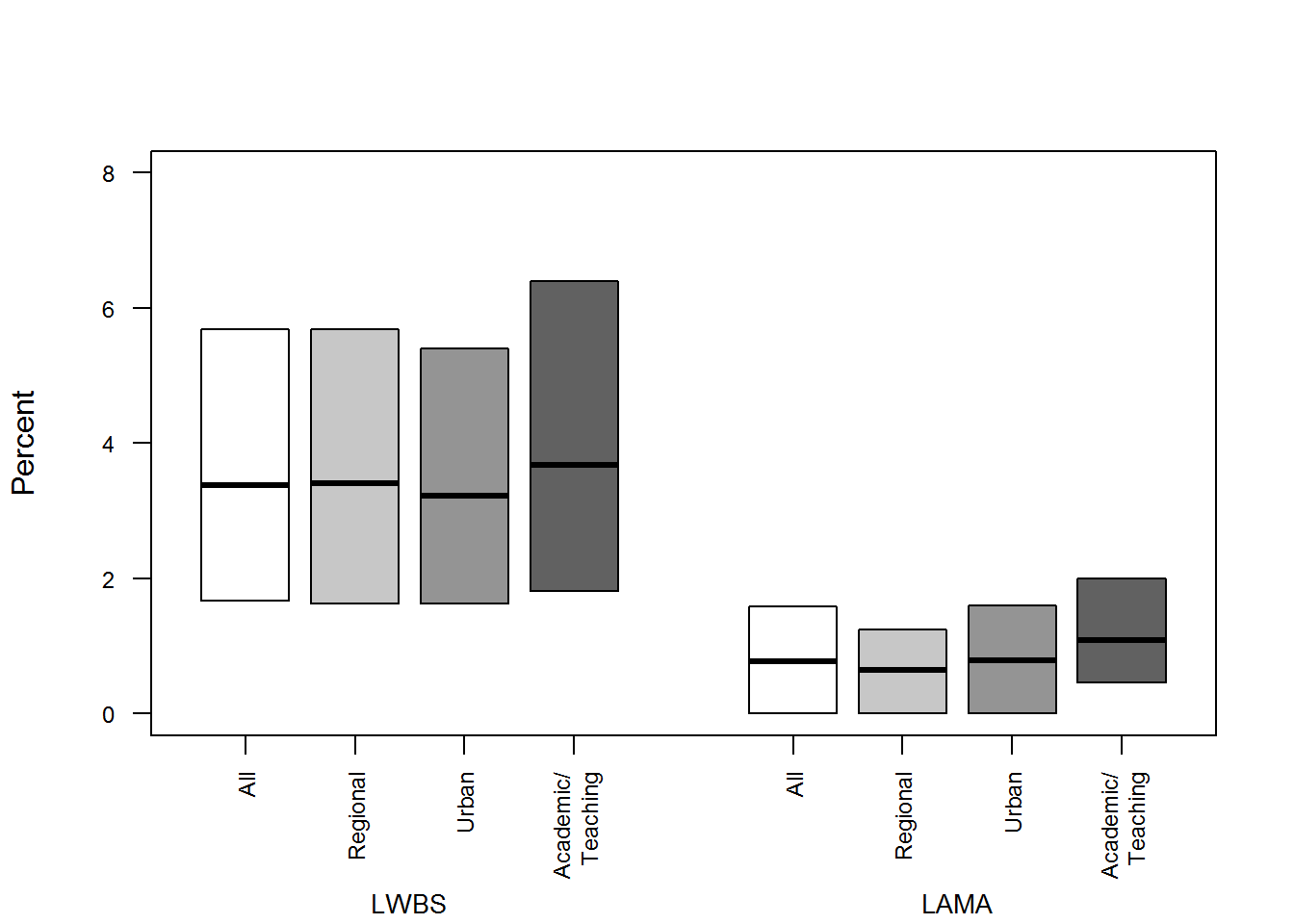


**eFigure S7.** Median and interquartile range (25th percentile, 75th percentile) for daily, facility-specific percent left without being seen (LWBS) by years and by ED category.


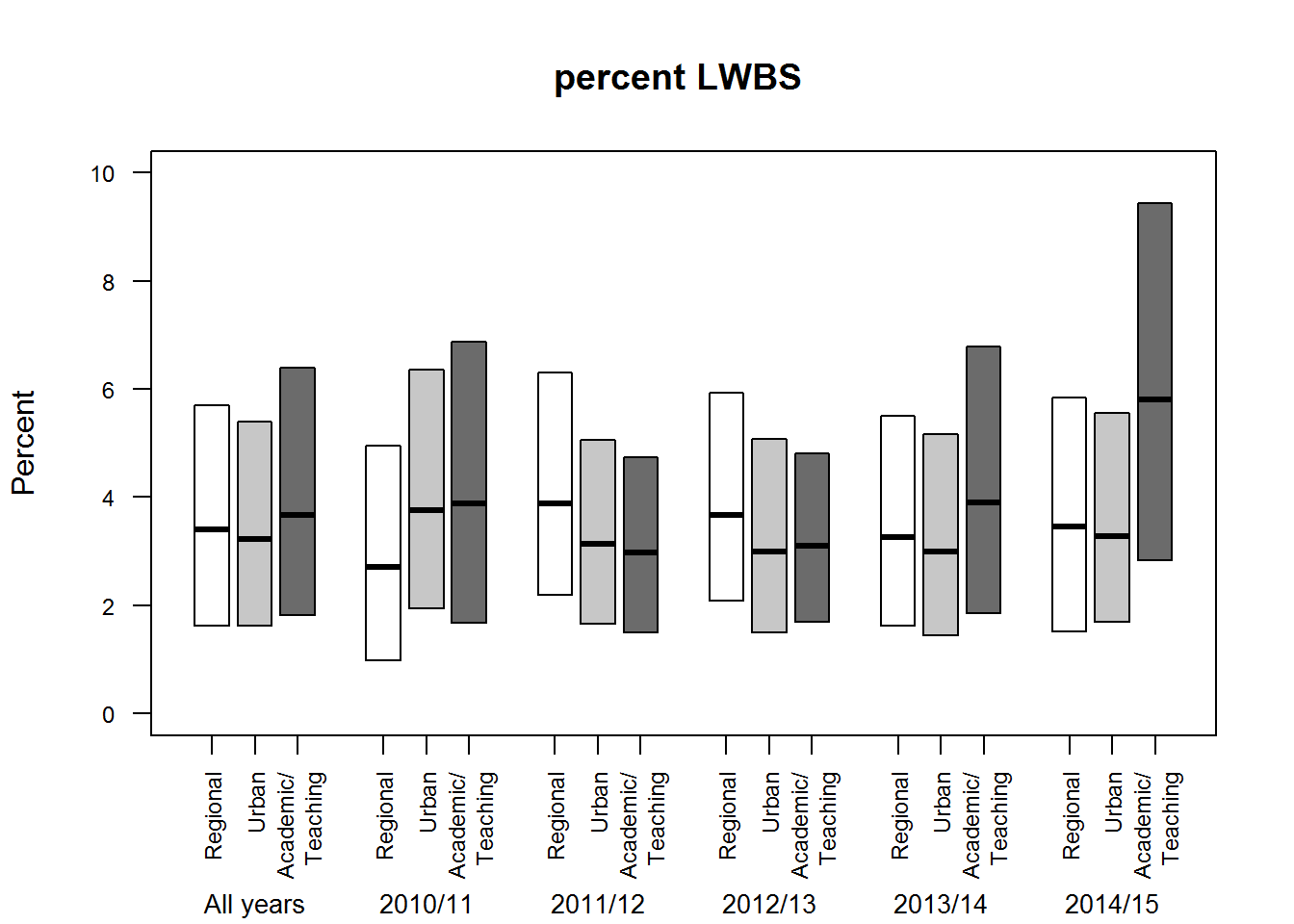


**eFigure S8.** Median and interquartile range (25th percentile, 75th percentile) for daily, facility-specific percent left against medical advice (LAMA) by years and by ED category.


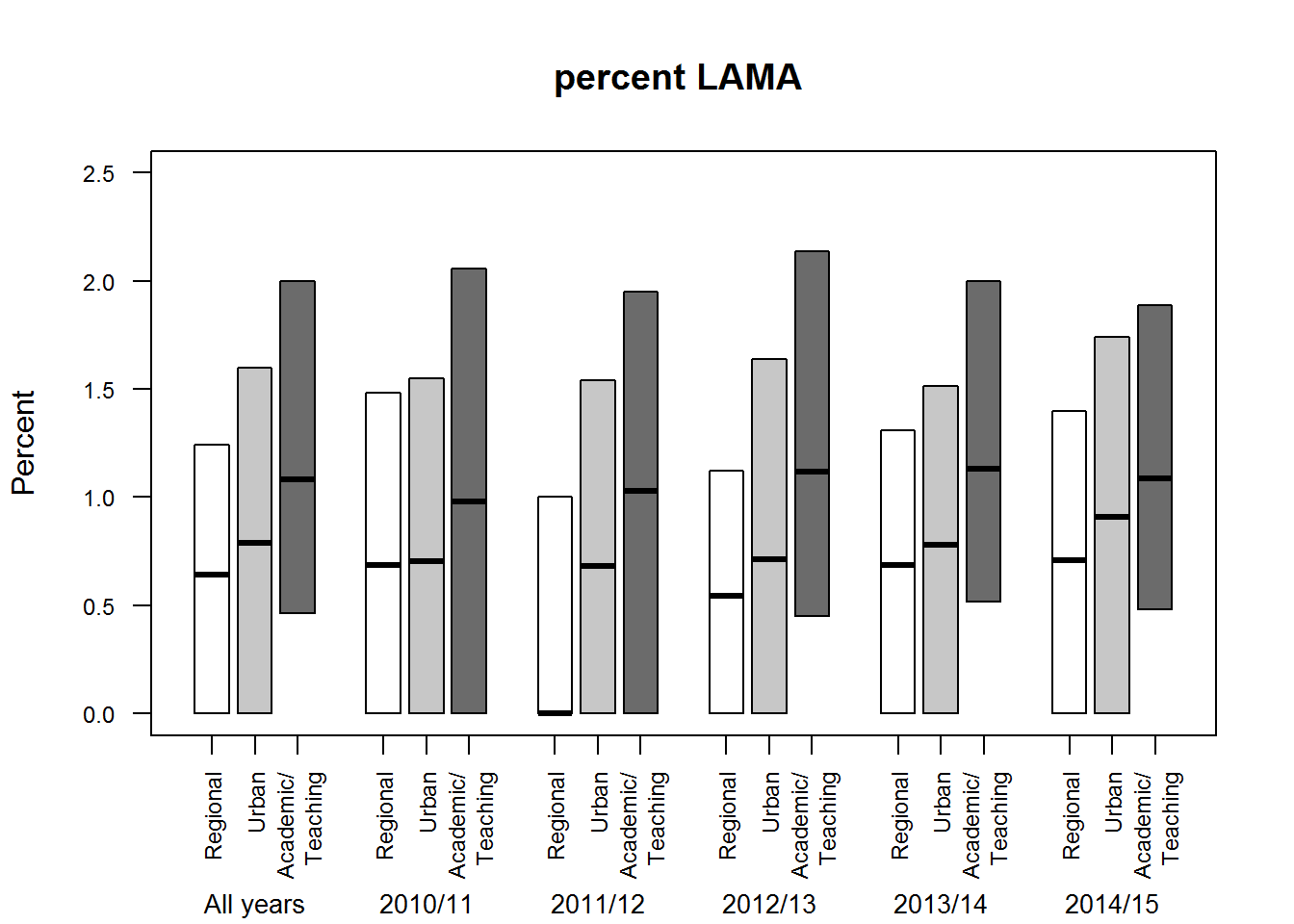

Supplement: Supplementary file 1 — Additional file 1: eTable S1. Percentages of presentations that exceed recommendations for all years and by fiscal year. eTable S2. Percentages of presentations that exceed recommendations by ED category for all years and by fiscal year. eFigure S1. Median and interquartile range (25th percentile, 75th percentile) hourly, facility-specific median physician initial assessment (PIA) times by years and by ED category. eFigure S2. Percent of presentations from all ED that exceeded the CAEP recommendations for medians for physician initial assessment (PIA), length of stay (LOS) for discharges with CTAS 1/2/3, LOS for discharges with CTAS 4/5, and LOS for admissions by ED category and by fiscal year (darkest grey 2010/2011, lightest grey 2014/2015). eFigure S3. Percent of presentations from all ED that exceeded the CAEP recommendations for 90th percentile for physician initial assessment (PIA), length of stay (LOS) for discharges with CTAS 1/2/3, LOS for discharges with CTAS 4/5, and LOS for admissions by ED category and by fiscal year (darkest grey 2010/2011, lightest grey 2014/2015). eFigure S4. Median and interquartile range (25th percentile, 75th percentile) for hourly, facility-specific median length of stay (LOS) for discharges by years and by ED category. eFigure S5. Median and interquartile range (25th percentile, 75th percentile) for hourly, facility-specific median length of stay (LOS) for admissions by years and by ED category. eFigure S6. Median and interquartile range (25th percentile, 75th percentile) of daily, facility-specific percent left without being seen (LWBS) and left against medical advice (LAMA) for all EDs and by ED category. eFigure S7. Median and interquartile range (25th percentile, 75th percentile) for daily, facility-specific percent left without being seen (LWBS) by years and by ED category. eFigure S8. Median and interquartile range (25th percentile, 75th percentile) for daily, facility-specific percent left against medical advice (LAMA) by [file 12913_2020_5196_MOESM1_ESM.docx]
